# Self-help for learned journals: Scientific societies and the commerce of publishing in the 1950s

**DOI:** 10.1177/0073275321999901

**Published:** 2021-03-18

**Authors:** Aileen Fyfe

**Affiliations:** University of St Andrews, UK

**Keywords:** Scientific journals, scientific societies, Britain, twentieth century, commerce, open access, academic publishing, scholarly communication

## Abstract

In the decades after the Second World War, learned society publishers struggled to cope with the expanding output of scientific research and the increased involvement of commercial publishers in the business of publishing research journals. Could learned society journals survive economically in the postwar world, against this competition? Or was the emergence of a sales-based commercial model of publishing – in contrast to the traditional model of subsidized journal publishing – an opportunity to transform the often-fragile finances of learned societies? But there was also an existential threat: if commercial firms could successfully publish scientific journals, were learned society publishers no longer needed? This paper investigates how British learned society publishers adjusted to the new economic realities of the postwar world, through an investigation of the activities organized by the Royal Society of London and the Nuffield Foundation, culminating in the 1963 report *Self-Help for Learned Journals*. It reveals the postwar decades as the time when scientific research became something to be commodified and sold to libraries, rather than circulated as part of a scholarly mission. It will be essential reading for all those campaigning to transition academic publishing – including learned society publishing – away from the sales-based model once again.

## Introduction

In 1895, the secretary to the Royal Society explained to the UK government that the publication of scientific research journals simply could not be undertaken “on an ordinary commercial basis.” This being so, “the burden” of publishing research necessarily fell on the learned scientific societies and their mission for supporting scholarship.^1^ Yet, just over a century later, the investment bank Morgan Stanley could describe scientific and medical journal publishing as an industry worth US$7 billion, offering good returns to investors.^2^ During the twentieth century, in other words, the publishing of research journals had become a lucrative business.

This transformation had two underlying components: a significantly increased involvement of commercial firms in journal publishing after 1945, and changing attitudes toward the economics of journal publishing among the learned societies. The dominance of modern research journal publishing by international media conglomerates has motivated many of the current campaigns for the reform of academic publishing.^3^ This paper, however, examines the shifts in thought and in practice that occurred at learned society publishers in the 1950s and 1960s.

The postwar decades were a key period of transition for learned society publishers: this was when they shifted from *circulating* research to *selling* it, and from decades of publication deficits to a new world of breaking even. Profits from publishing would be a later development (and not for all societies), but this was when scientific research became something to be commodified and sold to libraries, rather than circulated as part of a scholarly mission. Understanding the reasons for the 1950s shift from a philanthropic model of circulation to a commercial model of distribution based on sales is essential grounding for all those campaigning to transition academic publishing – including learned society publishing – away from the sales-based model once again.^4^ It also offers us a different perspective on the links between science and commerce during the late twentieth century.

## Commerce or mission?

Scientific journals have traditionally featured in the history of science as technologies that enable the construction and circulation of knowledge claims, and as sources of information about those claims, their authors, and their reception.^5^ More recently, historians have investigated how authorship in journals came to be such a dominant element in the prestige economy of scholarship, providing a route to reputation and potential professional advancement.^6^ The learned societies and academies of the eighteenth and nineteenth centuries are an important part of that story due to the tight connection they created between research publishing and communities of scholars. But we know remarkably little about how academic research journals developed from tools for circulating knowledge within communities of gentlemen-scholars into business enterprises generating substantial commercial rewards for their owners and shareholders.^7^ The rise of such firms as Pergamon Press, North Holland Publishing, and Elsevier (all now part of RELX Elsevier) in the mid to late twentieth century is familiar to industry practitioners, scholarly communications campaigners, and scholars of scientometrics, but it has yet to receive adequate attention as a historical phenomenon depending on, and significantly affecting, scientific research and the academic profession.^8^

The commercialization of scientific research publishing is intertwined with wider transformations of the scientific enterprise in the twentieth century: the increased scale of scientific research; increased funding, particularly in the early decades of the Cold War; international conferences and research collaborations; and the emergence of new and more specialized disciplines.^9^ Governments, philanthropic foundations, and industrial enterprises became more involved in funding research, and their willingness to fund the *publication* of research forms the backdrop to the commercialization of scientific journal publishing. More generally, academic scientists were also affected by changes in the funding and organization of higher education. For those based in the UK, that included adjusting from a British scholarly world, focused on the empire and Commonwealth, to one in which Europe and, especially, the United States were more prominent.^10^

One contemporary who was well aware of the business potential of international science was the publisher Robert Maxwell, the founder of Pergamon Press. Interviewed in 1964, he explained that “I believe that scientific research is an international thing not confined by national boundaries.” His approach to the publication of scientific research was therefore to be “speedy production and world-wide dissemination.”^11^ This offered a sharp contrast to learned society publishing, which was rarely described as “speedy” (partly because society editorial processes involved peer review, then known as “refereeing”).^12^ Although British learned societies certainly did think about dissemination beyond the UK, they typically focused on the needs of readers in the extended British academic world, with some recognition of traditional centers of scholarship in Western Europe.^13^ They had relatively little engagement with authors or readers in the United States, South America, Africa, East Asia, or the Soviet bloc.

In contrast, Pergamon Press drew upon the links Maxwell had developed with German publishers during his military service in postwar Berlin, and was internationally oriented from its foundation in 1951. Pergamon translated the research of Soviet scientists, published the proceedings of international conferences, and kept researchers on both sides of the Atlantic up to speed with each other’s work. It also created dozens of new journals to cater to emerging disciplines and specializations.^14^ Pergamon’s activities were partly responsible for the headline in *Nature* in 1960 that asked plaintively, “How many more new journals?”^15^

The rise of entrepreneurial, international scientific research publishing was not universally welcomed. A Royal Society committee noted in 1963 that “the present tendency for commercial publishers to initiate new scientific journals in great numbers is causing concern to many people.”^16^ Without mentioning names, concerns were voiced about the “less-scrupulous minority [of publishing firms] that act as *entrepreneurs*,” and who start new journals “in order to ‘sell’ science as a commodity.”^17^ There is no doubt that many scientists appreciated Maxwell’s support for themselves and their disciplines, but others believed he was “the greatest villain unhung.”^18^

The Royal Society was prominent among those who feared that entrepreneurial publishers posed a double risk to “the welfare of the scientific community.” In 1957 its executive secretary, David Christie Martin, explained that it was essential that scientific societies, rather than commercial firms, should “continue to predominate in scientific journal publication” because societies were committed, by their missions, to circulate research “as widely and cheaply as possible.” Furthermore, the expertise of their membership enabled them to ensure high intellectual standards and to be “the guardians of the quality of scientific publication of original work in learned journals.”^19^ The Royal Society’s concern was not, of course, disinterested: its long-established and well-respected role as a publisher of research journals was threatened by the ease with which commercial publishers were establishing influential new journals.

This existential threat arrived at a time when UK learned society publishers were already struggling with their financial sustainability. The situation had become so dire in the mid 1950s that contemporaries feared that, without some kind of help, learned society journals “were on the way to extinction.”^20^ This was not a uniquely British problem: in the United States, for instance, journals were facing such “critical financial problems” that it was a focus for the newly established National Science Foundation; its report led to changes that enabled U.S. government agencies to support the publication of research through the payment of page charges.^21^ In the UK, in contrast, there had been governmental support of learned society journals since the 1890s, but in austerity Britain of the early 1950s, the extent (or availability) of such funding seemed uncertain. It was in this context that the Royal Society urged learned societies to become self-reliant.

This paper explores the perceived threats to the survival of learned society publishers in the 1950s, and the strategies proposed to help them both adapt to the new world of postwar scientific publishing and free their journals from the prewar reliance on government support or charitable donations. It does so by examining a project intended to provide “Self-Help for Learned Journals” that ran from 1955 to 1963. This project was funded and supported by the Nuffield Foundation, but led by senior figures from the Royal Society, and it was intended to offer advice and funding to struggling learned society publishers.^22^ The project began with hopes of streamlining editorial and production processes to save money where possible, but it ended by urging the societies to focus on sales and marketing, especially internationally (and especially in the United States). Even though learned societies saw commercial publishers as a threat to the publication of research, the Self-Help project helped them learn how to think more commercially themselves.

## Learned journals and the threat of extinction

In the 1950s, learned society journals found themselves under such severe financial strains that it seemed “impossible” to continue circulating scientific research “on the old terms.”^23^ Those “old terms” had included a focus on wide circulation (for instance, sending free copies to educational and research institutions) and uneconomic pricing (for instance, discounted prices for members of the society and of other learned organizations).^24^ With so few copies being sold even at cost price, it is hardly surprising that learned society treasurers of the nineteenth century had been used to seeing journal publications as a drain on resources rather than a potential income stream.

Learned societies’ ability to fund the publication of ever-increasing amounts of knowledge had, in fact, been under strain for decades. This was why the UK government had agreed, in 1895, to provide an annual grant-in-aid of scientific publications, administered by the Royal Society and disbursed as grants to learned society publishers.^25^ This government support, plus some bequests and donations, and some rationalizations to the free or subsidized circulation, propped up the philanthropic model of learned society publishing in Britain until the Second World War.

In the immediate aftermath of the war, all members of the British print trades struggled to deal with the problems caused by wartime destruction and postwar shortages of materials and manpower.^26^ Even once the worst had passed, learned society publishers faced two specifically postwar problems. First, they could no longer rely on the “generous good-will” they had been accustomed to receive from printers, who faced their own challenges.^27^ In 1953, for instance, Cambridge University Press renegotiated terms with all learned societies for which it printed, including the Royal Society.^28^ Second, the cost of subsidizing the production and circulation of scientific research journals was constantly being pushed higher by the “sky-rocketing volume of original scientific publication.”^29^

It was in spring 1955 that the Nuffield Foundation and the Royal Society agreed to undertake a five-year project “for the support of learned journals.”^30^ The funding and clerical support would come from the Nuffield Foundation, while the Royal Society supplied expert advice. The Nuffield Foundation had been established by car manufacturer William Morris in 1943, with £10 million in shares and a remit to improve “social” or “industrial” medicine and scientific research in postwar Britain.^31^ Its desire to support capacity-building in science led to several collaborations with the Royal Society in the mid 1950s, most notably the Commonwealth scholarships.^32^ The stimulus for the learned journals project appears to have come from the Royal Society, but a parallel project was also established in collaboration with the British Academy.

As the oldest scientific society in Britain, the Royal Society liked to position itself as a leader among the newer, more specialized societies. It had led the appeal to government in 1895, and in 1948 it hosted international delegates at a Scientific Information Conference.^33^ In 1955, its approach to the Nuffield Foundation continued this leadership role, but also reflected the society’s own recent experience of transforming its journals from deficit to break-even and its doubts about the willingness of the UK government to continue supporting learned society journals.

The Royal Society had been forced to take a hard look at its own publishing practices in 1953. Between 1949 and 1952, the printed output of its *Philosophical Transactions* and *Proceedings* had risen from 3,700 pages to 5,460 pages a year, while production costs had risen even faster, from around £16,000 a year to over £35,000 a year.^34^ This forced the society to draw more heavily than usual upon its charitable reserves and upon the parliamentary grant-in-aid; otherwise, the society would have shown a publications deficit of £5,000 for the year ending November 1953.^35^ As the society’s officers debated the possibilities for cutting costs, a letter arrived from Cambridge University Press announcing that it had “reluctantly decided” that it could no longer continue “subsidising a number of learned Societies.”^36^ For the society’s officers, the possibility of having to pay a higher commission on the sales managed by the press put “a completely new complexion” on the situation.^37^

In less than a year, David Martin oversaw the creation of the Royal Society’s in-house publishing sales team, which took over sales, marketing, and subscription management from Cambridge University Press (CUP) in October 1954. (Printing remained at Cambridge, which was renowned for its high-quality scholarly typesetting.) The result was that sales, rather than philanthropy, came to be the main method of circulation for the society’s journals, and has remained so ever since. Severe cuts to the distribution of free copies meant that universities and research institutions that wanted to continue to receive the society’s journals henceforth had to purchase them. Prices were raised, and the sales team began to target new markets, particularly in North America. In 1953, Cambridge University Press had generated £27,500 of income for the society; by 1955, the society’s team reported a sales income of £58,500.^38^ This experience suggested to the Royal Society that learned society publishers could act to transform their precarious financial positions.

The further concern was uncertainty about the future of the parliamentary grant-in-aid for scientific publications. In the immediate postwar years, the UK government had been willing to accept that learned societies needed more help than ever with their journals and had increased the annual grant-in-aid from £7,000 in 1945 to £26,000 in 1954.^39^ But by the mid 1950s the political mood was changing, with chancellors from both dominant political parties implementing austerity measures. The grant-in-aid for 1957 would be reduced to £20,000, and it was against this context that David Martin advised the Chemical Society that it would be unwise to rely on government support for scientific publications. (The Chemical Society had received over £5,000 from the grant-in-aid for 1956.) Martin further hinted that “indebtedness” to government might even “endanger the independence” of the society and prevent it from being seen as “a strong force in the scientific community.”^40^ Martin’s success in transforming the Royal Society’s publishing finances made him unsympathetic to societies who continued to act as though “living in a fool’s paradise.”^41^ It was to help other societies become self-supporting that the Royal Society had sought the assistance of the Nuffield Foundation in 1955.

## The scientific advisory committee

The first meeting of the Nuffield Foundation’s “scientific advisory committee” was held on April 15, 1955. Its remit was to advise on the disbursement of £20,000 over five years, to assist learned journals in the sciences. The committee was dominated by the Royal Society: it was chaired by Edward Salisbury, the director of Kew Gardens and one of the secretaries to the society, and its original members included four other fellows of the society, and David Martin. The other committee members were Leslie Farrer-Brown, a medical administrator who had directed the Nuffield Foundation since its origin, and Jack Morpurgo, director of the National Book League, an organization founded in the 1920s to promote reading and an appreciation of books.^42^

At the first meeting of the scientific advisory committee, Farrer-Brown explained that the Nuffield Foundation intended its money to support what he termed “‘primary’ journals in ‘primary’ subjects,” a phrasing that the committee subsequently interpreted as journals publishing original research, with some bias toward the mathematical and physical sciences. The Nuffield Foundation appears to have shared David Martin’s conviction that societies should learn to help themselves, and laid down a guiding principle that support would be contingent on a journal showing it was “doing all within its power to make its economic position stable, or else is prepared to accept and act upon the advice offered by the panel of advisers.” Farrer-Brown explained that this condition was made to “encourage” societies “to put into practice possible economies in the production and distribution of their journals that may eventually lead to ensuring their solvency.”^43^ This emphasis on self-help was shared with the Royal Society committee that administered the parliamentary grant-in-aid, which, from 1957, required applicants to demonstrate that they were attempting “to stand on their own financial feet.”^44^

Unlike the parliamentary grant committee, which had to distribute all its funds to societies in need, the Nuffield committee had no such restriction. As Table 1 shows, by 1960 the Nuffield committee had spent only a quarter of its funds as grants to learned societies. Significantly, it had offered advice as well as money (and loans as well as grants), and it would attempt collective as well as individual solutions.

**Table 1.** Expenditure of the Nuffield scientific advisory committee, 1957–60.

Grants to society publishers	4,017
Loans to society publishers	5,860
Administration (consultant’s salary, secretarial assistance, expenses)	5,658
Other projects (creation of “select lists” of journals)	1,500
Repaid loans at October 5, 1960	–600
Total £	16,435

*Note*: These figures are for committed expenditure to October 5, 1960. Actual expenditure to that date was £16,136.

The first step taken by the Nuffield committee was fact-finding. Back in 1953, a meeting of society secretaries and editors had agreed that “there was a need for someone, knowledgeable both of the publishing trade and scientific journals,” to devote time to studying and advising learned society journals.^45^ The Nuffield committee consulted experienced publishers, including Allen Lane (of Penguin), Robert Lusty (of Michael Joseph Ltd), and Basil Blackwell (of Blackwell Scientific Publishing).^46^ Robert Lusty was then asked to pursue some informal enquiries, guided by a list brought to the first meeting by David Martin that classified twenty-five learned society publishers “from a financial point of view” (see Table 2).^47^ The Royal Society was one of just seven societies that Martin believed to be “successful,” but it subsequently emerged that the situation at the Chemical Society was not as rosy as he thought.^48^ Apart from the Chemical Society, all of the Nuffield committee’s help over the coming years would go to societies that were “in difficulties” or that did not even appear on Martin’s list.

**Table 2.** Scientific societies who publish journals (1955), with David Martin’s assessment of their financial status.

Successful	Biochemical Society *Chemical Society Faraday Society *Institute of Physics Institution of Electrical Engineers *Physiological Society *Royal Society
Borderline	Association of Applied Biologists Cambridge Philosophical Society *Company of Biologists
In difficulties	Botanical Society of the British Isles British Glaciological Society *Linnean Society London Mathematical Society Malacological Society Mineralogical Society Physical Society Royal Anthropological Institute Royal Astronomical Society Royal Entomological Society *Royal Meteorological Society *Royal Microscopical Society Royal Society of Edinburgh Society for Endocrinology Zoological Society

This list (originally in alphabetical order) was appended to the minutes of the first meeting of the Nuffield scientific advisory committee, LJ-SAC, April 15, 1955, Nuffield archives L.J.1. The asterisks indicate societies approached by Robert Lusty for his investigation.

Lusty was advised to talk to representatives of eight societies, representing a spread of disciplines and financial situations, and he reported back in March 1956.^49^ He examined three aspects of learned journal publishing: administration, production, and distribution. He was clearly surprised to see so much “slogging clerical work” being done by “professors and scientists,” and saw few opportunities for easy savings in the editorial and administrative processes. As *Self-Help* later put it, “nobody ever got paid any money for anything.”^50^ As for production, Martin hoped that a technical revolution in printing might significantly reduce costs, but Lusty felt that no “escape from letterpress” printing was likely in the foreseeable future. He admitted that minor economies in production might be achieved, but he saw no simple or single solution.^51^ With no administrative overheads to trim, nor any real hope of significant cuts in production costs, Lusty concluded that the only answer was to find ways of generating more income. This would become the key element of the self-help strategy for scientific journals.

Lusty had suggested the creation of a voluntary organization to enable societies to work together and share best practice, but the members of the Nuffield committee felt that the societies lacked the necessary “habit of co-operation.” Instead, they decided to hire a “liaison officer” to offer tailored advice to individual societies “to help them increase efficiency and revenue.”^52^ As Table 1 shows, this was a considerable expense (and one that would not have been possible under the terms of the parliamentary grant-in-aid), but it was central to the committee’s approach. The consultant spent many hours in “detailed discussions with editors and treasurers about their problems.”^53^ Even societies deemed too provincial or too niche for financial support could be offered advice.

The committee’s ideal candidate was someone who could move between the worlds of publishing and science, and who would “get on well with the editors of learned journals and officers of learned societies. He would have to be a man with general promotional and administrative ability and a sufficient contact with science not to blunder. It would be an advantage if he had a degree in science and some acquaintance with research.”^54^ Their first assistant met only part of this brief: Charles Hutt would later be described as “one of the leading science publishers of his generation,” but he was not a scientist.^55^ He had been responsible for developing the scientific publishing division at Butterworth & Co., following the 1951 dissolution of a short-lived Butterworth-Springer collaboration (brokered by Maxwell).^56^ In mid 1956, Hutt began discussions on behalf of the Nuffield committee with the Royal Astronomical Society, the Royal Anthropological Institute, the Royal Meteorological Society (all “in difficulties,” see Table 2), and the Chemical Society.^57^ However, the committee soon became uneasy about a potential conflict of interest. Hutt had offered to undertake his preliminary investigations “without remuneration,” but he was “perfectly frank about his commercial aspirations.” He would shortly move to Pergamon Press, and subsequently to Academic Press, and appears to have been too enmeshed in the world of commercial science publishing to “get on well” with the academic editors and officers of learned societies. In mid 1957, the committee tactfully dispensed with his services.^58^

Fortunately, another candidate emerged. An American by birth, Frank V. Morley (1899–1980) had recently retired from a long transatlantic publishing career that had included a directorship at Faber & Faber (with T. S. Eliot) in the late 1920s, and a senior role at Harcourt Brace in New York during the war years. He also happened to be a former Rhodes scholar with a D.Phil. in mathematics from Oxford.^59^ This made Morley a very plausible “liaison” between the worlds of publishing and science. In March 1957, Edward Salisbury reported that he and Martin had been “favourably impressed” with Morley, and believed he “had the right approach.”^60^ Morley would work for the Nuffield Foundation until at least 1961, and would describe his work in the 1963 booklet *Self-Help for Learned Journals*.^61^

A short article in *Nature* in September 1957 carefully introduced Frank Morley to the wider scientific community as someone who would be sympathetic to learned societies and scholars. As well as his own credentials, Morley’s family connections proved useful: he had two brothers in literary and academic roles in the United States, and a father who was a mathematics professor and journal editor. According to *Nature*, this meant that Morley had been “familiar with and interested in the problems attendant on the production and distribution” of his father’s journal “from an early age.” He was presented to academics and editors of learned societies as someone whose “wide and varied experience” might “prove of practical value,” but, equally importantly, as someone who understood their world.^62^

Overall, Morley engaged with at least twenty-eight societies, fifteen of which received some form of financial assistance. Table 3 lists all those societies recorded in the committee minutes as receiving some form of help, whether of money and advice (Table 3a) or advice only (Table 3b).^63^ Morley began with the societies on Martin’s 1955 list, but later reached out to some of the larger provincial societies and to smaller societies in more specialized fields. As the committee’s work became better known, it also began to receive unsolicited applications. Few of these were deemed eligible for funding, but the committee was willing to offer Morley’s advice to anyone who sought it, including the editor of *Business History*.

**Table 3.** Societies helped by the Nuffield scientific advisory committee, 1957–60. **Table 3a.**. Societies that received financial assistance as well as advice.

Society	Date helped	Funding (£)	Comment
Botanical Society of the British Isles	February 24, 1959	50	Grant for promotion scheme (had been receiving advice since February 17, 1958)
British Glaciological Society	December 4, 1957	250	Grant for “the preparation and distribution of a leaflet for the *Journal of Glaciology*”
British Herpetological Society	September 8, 1958	60	Grant for publicity in United States
Chemical Society	July 8, 1957	500	Grant for mailing list for UK and overseas, and a leaflet
Edinburgh Mathematical Society	October 5, 1960	1,000	Loan for reprinting (committee had declined financial assistance February 17, 1958, but later agreed)
Geological Society	October 28, 1957	250	Grant for publicity campaign for *Quarterly J* and for *Memoirs*
Linnean Society	February 24, 1959	600	£600 loan to fund production of microfiche edition of the Linnean herbarium, for overseas sales. The repaid loan was reissued (March 2, 1960) as a grant for a promotion scheme for the microfiches
London Mathematical Society	February 17, 1958	2,550	£250 grant for promotion scheme, and £2,300 loan for reprinting back numbers
Mineralogical Society	March 2, 1960	220	Grant for promotion scheme (originally offered £150, but increased upon request to allow a more elaborate leaflet)
Palaeontological Society	May 18, 1960	250	Grant for promotion scheme
Royal Anthropological Institute	May 29, 1957 December 4, 1957	225 225	Grant (in two parts) for publicity aimed at new members, libraries, conference delegates, and members of the American Anthropological Association
Royal Astronomical Society	March 25, 1957 October 28, 1957	300 200	Grant for a “publications sales drive” (but only £157 claimed) Grant for a prospectus for the new *Geophysical Journal*
Royal Meteorological Society	October 28, 1957 June 23, 1959	600 1,960	Grant for promotional scheme for two journals (inc. United States) Loan for reprinting back numbers from 1945
Society for Applied Bacteriology	May 18, 1960	100	Grant for promotion scheme in United States (but had asked for £250)
Yorkshire Geological Society	October 9, 1959	80	Grant for promotion scheme (had been receiving advice since February 24, 1959)

**Table 3b.** Societies that only received advice.

Society	Date	Comment
Bee Research Association	October 5, 1960	“No question of a grant in this case”
British Bryological Society	February 17, 1958	Morley had preliminary talk
*British Journal of Herpetology*	June 23, 1958	
*Business History*	June 23, 1959	Not eligible for grant, but Morley can offer advice
Institute of Navigation	June 23, 1958	
Liverpool & Manchester Geological Society	February 24, 1959	
Malacological Society	February 17, 1958	Meeting to be arranged (February 24, 1959: Morley has talked to them about pricing)
Mathematical Association	September 23, 1957	
Pharmaceutical Society	September 23, 1957	Refused a grant, because it “should be able to pay its way”
*Quarterly Journal of Experimental Physiology*	February 24, 1959	Sought help with promotion scheme; committee wanted to see detailed accounts before offering funding
*Royal Microscopical Journal*	October 9, 1959	“A worthy journal,” Morley can offer advice
Royal Society of Edinburgh	February 17, 1958	Morley to have preliminary talk
South London Natural History Society	June 23, 1958	“Could not make ends meet” (but not considered “primary”)

The interest from smaller societies contrasted with Morley’s early difficulties persuading the larger societies to participate. There was some reticence about sharing financial details with outsiders, as well as a fear of forcible “interference with editorial policy or any lowering of standards.”^64^ Morley had to reiterate that he and the committee would only “consider financial matters, such as (1) price of journal; (2) need for new subscribers.”^65^ Even so, Salisbury would later note that there had been resistance from “those who had, under other economic conditions in the past, given ungrudging and successful service as editors and members of editorial boards.”^66^ The implication is that many scientists did not (yet) fully appreciate the changed economic realities of the postwar publishing landscape and were hoping that journal publishing could, somehow, keep going as before.

As a general rule the Nuffield committee helped learned societies, but one exception was the *Quarterly Journal of Experimental Physiology*, whose editor applied for assistance in 1957. Although the journal was “published by Livingstones in Edinburgh, who act as distributors,” its ownership was vested in three (academic) trustees.^67^ The committee encouraged the editor to talk to Morley, but when he subsequently applied for a grant, he was informed that “there was really no good case.” The reasons were twofold: the journal’s publisher had agreed to contribute toward the cost of the proposed publicity campaign, but most crucially (and apparently thanks to Morley’s initial advice), the journal was now making a small profit.^68^ The Nuffield committee was apparently willing to take a generous definition of a “learned journal,” but its financial support was limited to those (still) “in difficulties.”

As the project wore on, Morley and the committee became increasingly conscious of the various relationships between learned societies and the firms who provided services to them. For instance, in 1957 a grant to the Geological Society was made on condition that the publicity material clarified that the printer “was acting solely as the printer, on behalf of the Geological Society, and would derive no financial gain from the resulting sales.”^69^ Later, it investigated the “exact contractual arrangements” between the London Mathematical Society and its printer.^70^ The variety of arrangements it discovered would be demonstrated by the three pages of examples included in *Self-Help*.^71^ For societies that paid for publishing (as well as printing) services, sales-based commissions made it tricky for the Nuffield committee to help the society with sales and marketing without also helping its publisher; in such cases, the committee expected the publisher to contribute to the costs of any publicity campaign from which it would also benefit.^72^ The ambivalence about working with profit-seeking partners can be seen most clearly in the Royal Meteorological Society’s proposed reprinting project: its printer was willing to underwrite the project, but the society preferred to take a loan from the Nuffield committee so it could retain its ownership rights and “remain independent.”^73^

The Nuffield committee entirely declined to support ideas that had clear scholarly value but little apparent chance of commercial success. For instance, the *Journal of Animal Ecology* was informed that index-making could only be supported if “there was any indication that the Journal would improve its finances” as a result.^74^ As the next section will show, the committee’s support was largely directed toward helping learned societies run their journals on more commercial lines.

## Learning to self-help

Morley began *Self-Help* with a discussion of cost-saving opportunities, but it was a notably brief section. As Lusty had earlier observed, it was difficult to find simple changes that would dramatically improve a society’s publishing finances. Morley pointed out that a change of page size might reduce postal charges, or make it easier to accept adverts, but he was wary about suggesting changes that might be seen as lowering a society’s traditional production values. His most concrete recommendation was for those societies whose journals mixed original research articles with such content as the annual accounts, reports of meetings, and members’ news. Removing such “parochial” material would save paper and postage costs, and make the journal more attractive to overseas (and nonmember) subscribers. Thus, the British Glaciological Society created a members-only bulletin separate from its research journal.^75^

Apart from that, Morley reminded academic editors and their assistants that they could avoid many delays and expenses if they would take the time to learn how a printing office really worked. Editors who understood that the printer has “to satisfy other customers who are on time” would realize why punctuality in returning proofs mattered, and understanding how corrections were made to typeset text would reveal why authors “should be utterly forbidden” the “ancient luxury” of rewriting papers at proof stage.^76^

Most of the pages of *Self-Help*, however, and most of the discussion in the committee minutes, focused on ideas for revenue-generation. They reveal the steps British learned societies took as they transitioned to a more commercial outlook for their publishing activities.

### Prices and marketing

The pricing strategies of learned societies had developed in the age of independent gentleman scholars, with the underlying assumption that researchers would access journals by acquiring personal copies. Those who were members of a particular society typically got the society’s journal free or discounted, and, even for nonmembers, the societies set prices low to enable wider circulation. For instance, in the early twentieth century the Royal Society had offered discounted rates on its journals to members of other scientific societies and kept its official nonmember price low for the benefit of younger scientists who were not (yet) members. Lusty had been swift to label such pricing practices as “hopelessly uneconomic,” and Morley was scathing of society members who expected “to be supplied at prices fixed before the First World War.” In an age of inflation, the simplest way to prevent societies “slipping into insolvency” was to raise prices.^77^

By the 1950s, very few nonmembers were buying personal copies. Most scientific researchers now had access to institutional libraries affiliated to their university, industry, or military laboratories. Nonmember prices had been set low to support early-career scientists, yet it was actually libraries that were “almost the only other purchasers” (other than society members).^78^ This was why Pergamon Press adopted a subscription system that differentiated between individual and institutional purchasers.^79^ Morley did not suggest societies should replace their traditional member/nonmember distinction, but he did argue that both rates must be economic.

In *Self-Help*, Morley used circulation figures from the (anonymized) Chemical Society to demonstrate the effects of a series of price rises during the 1950s.^80^ In 1949, a subscription to its journal had cost £1.10s for members, and £5 for nonmembers. Ten years later, the rates were £8 and £20 respectively. The dramatic increase in price for society members hit hard: over 75% of the UK-based members stopped taking personal copies of the journal. On the other hand, a successful marketing campaign aimed at libraries increased the number of nonmember subscribers by almost 50%. Overall, the total circulation decreased by 900 copies, *but* the journal became financially self-supporting.^81^

The story encapsulates the transition from the philanthropic to the commercial mode of scientific journal publishing: the journal was now funded by library subscribers rather than subsidized by grants; its readers relied on library copies rather than their own personal copies; and the market became international rather than British. Morley argued that each society “must strike its own balance” between how far “it can afford to cosset its own members” and how far “to seek commercial return from non-members,” but he insisted that “the economic pressure is ruthless.” He saw this as “part of the whole social change” in postwar science, and the postwar world more generally.^82^

The Chemical Society’s new financial stability illustrated the importance of a successful marketing campaign, as well as higher prices. Almost all the grants made by the Nuffield committee were for the printing and mailing costs of promotional campaigns, and it was their design and implementation that occupied most of Frank Morley’s time in late 1957 and 1958. The biggest cost in any publicity campaign at this time was the postal charges, so it was essential that any material sent out was effective and that it was sent to the right people.

Morley worked with societies to design their promotional material, usually recommending a two- or four-page leaflet that could be used to “present the right ‘image’” of a journal. For instance, he advised the Geological Society on “a more effective” layout and advised the Mineralogical Society on “a rather more elaborate leaflet. . ., including perhaps a plate.” Morley insisted that it was worth “spending a little to produce a better leaflet.”^83^

When it came to circulating this publicity material, Morley repeatedly argued that “head-work” was more important than “unguided promotional efforts,” and that learned societies had – in their own membership – excellent resources for selecting and scrutinizing a specialist mailing list.^84^ It was possible to hire an agency to send promotional material “to ‘a good list’ of 5,000 addresses or more” – but for specialized scientific journals, this was unlikely to be money well spent.^85^ The Royal Astronomical Society had initially sent a leaflet promoting its *Monthly Notices* “to a list supplied by the printer,” and although it told the Nuffield committee that the results had been “less disappointing than was feared at one stage,” they subsequently planned to send the leaflet to “a hand-picked list of 500 scientific and national libraries and institutions.”^86^ The Chemical Society, too, created a “hand-picked” list, though its focus was “1,500 industrial addresses.”^87^ Morley explained that he encouraged society officers to use “their own knowledge” to identify “individual heads of departments at American universities and colleges”; this was far better than using standard lists of American libraries because “librarians are shy birds to shoot at.”^88^

This focus on overseas, and especially North American, markets was a change of approach for British learned societies, which had traditionally focused on their British-based members and on scientific institutions in the British sphere of influence. It reflected a widespread postwar awareness of the importance of international scientific communication, within (and perhaps beyond) the NATO bloc.^89^ Most societies applying for Nuffield help were interested in the “considerable untapped market in America.”^90^ Shared language made the United States an obvious target for British publishers, but before the war, both the book trades and the academic worlds of the United States and the UK had been only loosely connected. The creation of the Fulbright scholarships in 1946 and the opening of a U.S. branch office by Cambridge University Press in 1949 were among the signs of change.^91^ The Royal Meteorological Society, the Palaeontological Society, and the Society for Applied Bacteriology were among those who received funding specifically for publicity campaigns in the United States.^92^

The Nuffield committee also encouraged the sharing of resources. For instance, from September 1958 it began collecting specimen mailing lists from societies seeking grants, and making them available for consultation by other (approved) societies.^93^ Thus, in 1960 Morley was able to improve the mailing list drafted by the Yorkshire Geological Society by drawing on lists compiled by the Geological Society and the Royal Society.^94^

Morley also coordinated a collaboration with the British Council. Its offices overseas were to supply lists of British scientific journals to “scientists, science libraries and bookshops in many countries,” hopefully benefiting many societies by “extending sales and the use of these periodicals overseas.”^95^ The Nuffield committee members were enthusiastic about this plan, but progress was slow as the organizations involved bickered over who should take responsibility for compiling and – even more critically – publicly endorsing the selected, annotated lists of journals. It took more than two years before the first such list – for geological journals – was in print.^96^

### Other revenue: advertising and “hidden assets”

Many editors were interested in the potential of income from advertising, but it would prove elusive. Early in the project, Frank Morley contacted the general director of Shell-Mex and BP (the joint UK marketing venture of Royal Dutch Shell and British Petroleum), and discovered that he was certainly interested in bringing his company to the attention of researchers in the geological and geophysical sciences, if it could be done without having to deal “separately with individual journals.”^97^ Morley therefore tried to persuade the major geological journals to work together so that they could, as a group, enter an advertising contract with Shell-Mex and BP. This “group advertising” would make the logistics easier for the advertiser as well as offering access to a larger combined audience. As Morley later put it, such “a really wide coverage of alert minds” ought to be attractive to the sort of scientific and technical firms that sought to recruit (or sell to) “the best and best-trained brains in the country or overseas.”^98^ He hoped that Shell-Mex and BP would be the first of many large industrial advertisers for British scientific journals.

By February 1958 the scheme had been “tentatively organised,” but it never progressed further.^99^ There were some issues from the Shell-Mex end, but the real problem lay with the geological societies. A practical sticking point was that, for such a scheme to work effectively, journals would need to coordinate certain elements of their production processes, such as page size and periodicity, but these were significant changes to make for an as-yet-uncertain (but certainly modest) financial benefit. Morley reported in 1959 that only the “smaller societies” had shown much interest.^100^ He later described the challenges of getting societies to work together – and particularly, persuading the larger societies to work with smaller organizations – as a difficult “psychological problem,” akin to persuading lions “to lie down with lambs.”^101^ The abortive group-advertising scheme demonstrates one of the big challenges for the Nuffield scientific advisory committee: individual societies certainly wanted help, but were not keen to work together.

A more fruitful mechanism for income-generation turned out to be the exploitation of “hidden assets” or “buried treasure”: these were assets owned by the society that “may be profitably reproduced.”^102^ Journal back-runs were a case in point, as the successful publicity campaigns attracted the attention of libraries that might be persuaded to purchase the recent back-run as well as current and future issues. In 1957, for instance, the Company of Biologists “made a big profit” on the sale of back numbers of its journal.^103^ This plan, however, depended on having complete sets to sell, and most societies had gaps in their warehouse holdings. This was why the Royal Society embarked on a reprinting program to generate sets of its *Proceedings A* (physical sciences) from 1939–56. By 1959 it was reporting “very encouraging” sales, but the “considerable” upfront costs would be a barrier to most societies.^104^

This was where the flexibility available to the Nuffield scientific advisory committee proved its worth: from spring 1958, it began to offer interest-free loans to societies (see Table 3a). The scale of funding needed for reprinting projects is clear in the higher value of such loans (£1,465 on average, compared to £260 for grants), and explains why the Nuffield committee was unwilling to make grants for these projects. Three of the loans were made for reprinting journal back-runs, but the fourth loan (and the only one that had been repaid by October 1960) was to help the Linnean Society create microfiche sets of its collection of dried botanical specimens “for sale throughout the world.”^105^ A year later, its sales were said to be “going very well.”^106^ Extra revenue from the exploitation of “hidden assets” did not change the long-term sustainability of a society’s day-to-day journal publishing operations, but it could be used to plug any remaining gaps in the publication finances in the short term, or as capital to support innovation.^107^

For modern academic authors, the absence of any discussion of page charges may seem striking. In the United States at this time, some learned societies were indeed trying to improve the financial situation of their journals by asking authors to contribute to the cost of publishing their articles. The American Institute of Physics had been doing it since the 1930s, and in 1954 the National Science Foundation reported that at least sixteen society publishers were using page charges.^108^ It became even more common in the 1960s and appears to be linked to the willingness of private funders and U.S. government agencies to pay such charges on behalf of their authors.^109^ The absence of page charges from the UK debates presumably reflects the different history of government funding for scientific research, and a different pre-existing mechanism for supporting its publication.^110^ It is unclear how much British and American learned societies knew about each other’s practices.

## Conclusions

By 1960, the Nuffield Foundation’s scientific advisory committee had reached the end of its original terms of reference. Frank Morley submitted his report to the eighteenth and final meeting in October that year, and was thanked by the chairman, Edward Salisbury, for his “valuable work.”^111^ There was a general sense that the committee’s efforts had been successful, and its work would be taken forward in two ways. First, Morley continued to work for the Foundation, overseeing the completion of the “select lists” of journals for distribution by the British Council overseas, offering advice to more societies, and transforming his private report into *Self-Help for Learned Journals* (1963). Second, some of the other committee members continued to engage with the problems facing learned journals through the Royal Society’s “scientific information committee.” For instance, in 1963 the Royal Society hosted a meeting of representatives from fifty-five UK learned societies at which a draft of *Self-Help* was circulated to stimulate discussion about the problems of scientific publishing; this led to an annual Conference of Editors throughout the 1960s.^112^

Salisbury’s preface to *Self-Help* praised the Nuffield committee’s intervention as a “major service to scientific publication in this country” that had resulted in “vistas of growing deficits” being turned into “prospects of financial stability.”^113^ Morley was more down-to-earth, writing that it was “a success story – if only to the extent that none of the patients died.”^114^ Few traces survive of the financial details of the societies that received assistance, though copies may survive in the societies’ own archives. For instance, in January 1958 the honorary secretary of the Royal Anthropological Institute (originally classified as “in difficulties”) reported that the sales income from its journals had been greater than in any previous year. She was unwilling to attribute this improvement entirely to the Nuffield grant for a promotion drive, but she did allow that “some portion of this is due to their assistance and is surely in the right direction!”^115^ Eighteen months later, the Nuffield committee was pleased to learn that the Royal Anthropological Institute’s annual publication deficit of over £2,000 in 1956/7 had been reduced to just £17.^116^ That a deficit of £17 was seen as a success reminds us that the aim was sustainability, not – in contrast to later decades – profit.

The Nuffield scientific advisory committee’s success in persuading learned societies to analyze and adjust their journal publishing practices was due partly to its ability to draw upon the Royal Society’s institutional and personal networks, and partly to its appointment of Frank Morley. Morley’s personal experience and background was key, but the very decision to hire a consultant, and offer transformative advice as well as money, distinguished the scientific advisory committee’s approach from that of the parliamentary grant-in-aid committee, various private charities, and even (apparently) the Nuffield Foundation’s British Academy learned journals committee.

Learned society journals did not, therefore, go extinct in the “lean years” between 1955 and 1962.^117^ Their survival has meant that, although academic journal publishing in the early twenty-first century has come to be dominated by just four international media conglomerates, those four firms do not dominate the natural sciences as much as they do (for instance) the social sciences.^118^ Scientific learned societies in both the UK and the United States remain important journal publishers, alongside the big commercial firms. It is true that the most significant UK society publishers – the Institute of Physics and the Royal Society of Chemistry – were among those that were already designated “successful” in 1955, but the Nuffield project enabled many smaller society publishers to survive.

Robert Maxwell created Pergamon Press to thrive in the conditions of postwar scientific research, with its expanding number of specialist fields, the international scope of its collaborations and communities, and its well-funded laboratories and libraries. Older journal publishers, in contrast, had to learn how to adapt their existing practices to the changed conditions. This was true of both older firms, such as Taylor & Francis and Wiley, and the learned society publishers. But the transition was more dramatic for the learned societies, as it involved reinterpreting their mission to circulate the results of scientific research as widely as possible. Rather than being seen in largely philanthropic terms, journal publishing came to be seen as a commercial enterprise – even at learned societies whose officers warned of the potential dangers of undue commercial influence.

It is a change that has had long-term consequences for learned society publishers. Becoming self-supporting turned out to be not only a solution to the immediate existential threat, but the first step toward making profits. By the later twentieth century, publishing income enabled learned societies to support a variety of activities – from policy work, to research grants, prizes, and the promotion of science in schools – that would have been utterly impractical for their nineteenth-century predecessors.^119^ An unwillingness to abandon such work underpinned the initial wariness displayed by many learned society publishers toward the open access movement of the early twenty-first century.

This paper demonstrates that a sales-driven, profit-seeking commercial approach is not a natural or essential element of scientific journal publishing. It has come to dominate journal publishing only since the 1950s and 1960s, and it did so in very particular politico-economic and technological circumstances, not all of which still pertain in the early twenty-first century. This paper also reminds us that even organizations with proud histories and engrained practices can adapt successfully to changed circumstances. whether an existential threat, similar to that facing learned society publishers in the 1950s, is a necessary stimulus.

## Notes

1. Lord Rayleigh, for the Royal Society, to H.M. Treasury (approved draft), June 20, 1895, in Royal Society archives, London (hereafter RS) Council Minutes (Printed) (hereafter CMP) CMP/07.

2. Morgan Stanley Equity Research, *Scientific Publishing: Knowledge Is Power* (London Morgan Stanley, 2002), p.2.

3. Vincent Larivière, Stefanie Haustein, and Philippe Mongeon, “The Oligopoly of Academic Publishers in the Digital Era,” *PLOS ONE* 10 (2015): e0127502.

4. For instance, the work of the Society Publishers’ Coalition (founded 2018, mostly UK/EU-based) and Transitioning Society Publications to Open Access (founded 2018, mostly U.S.-based). In 2019, the Wellcome Trust (with the Association of Learned and Professional Scholarly Publishers) funded a consultancy project to help society publishers accelerating open access, in a move with some similarities to the Nuffield Foundation/Royal Society project.

5. Steven Shapin, “Pump and Circumstance: Robert Boyle’s Literary Technology,” *Social Studies of Science* 14 (1984): 481–520; Adrian Johns, “Miscellaneous Methods: Authors, Societies and Journals in Early Modern England,” *British Journal for the History of Science* 33 (2000): 159–86; Alan G. Gross, Joseph E. Harmon, and Michael S. Reidy, *Communicating Science: The Scientific Article from the Seventeenth Century to the Present* (New York: Oxford University Press, 2002).

6. Alex Csiszar, “How Lives Became Lists and Scientific Papers Became Data: Cataloguing Authorship During the Nineteenth Century,” *British Journal for the History of Science* 50 (2017): 23–60; Alex Csiszar, *The Scientific Journal: Authorship and the Politics of Knowledge in the Nineteenth Century* (Chicago: University of Chicago Press, 2018); Melinda Baldwin, *Making “Nature”: The History of a Scientific Journal* (Chicago: University of Chicago Press, 2015).

7. My focus here is specifically on those periodicals that focus on the publication of original research claims. Many other sorts of periodicals publish scientific content, and commercial interests were clearly present in some genres long before the twentieth century; see, for instance, William H. Brock, “The Development of Commercial Science Journals in Victorian Britain,” in A. J. Meadows (ed.), *The Development of Science Publishing in Europe* (Amsterdam: Elsevier, 1980); Jonathan R. Topham, “The Scientific, the Literary and the Popular: Commerce and the Reimagining of the Scientific Journal in Britain, 1813–1825,” *Notes and Records* 70 (2016): 305–24.

8. A. J. Meadows (ed.), *The Development of Science Publishing in Europe* (Amsterdam: Elsevier, 1980); Robert Campbell, Ed Pentz, and Ian Borthwick (eds.), *Academic and Professional Publishing* (Oxford: Elsevier Science, 2012); Michael Mabe and Anthony Watkinson, “Journals (STM and Humanities),” in Andrew Nash, Claire Squires, and Ian Willison (eds.), *The Cambridge History of the Book in Britain, Volume 7. The Twentieth Century and Beyond* (Cambridge: Cambridge University Press, 2019), pp.484-498; Larivière, Haustein, and Mongeon, “The Oligopoly of Academic Publishers” (note 3).

9. For instance, Peter Galison and B. W. Hevly, *Big Science: The Growth of Large-Scale Research* (Stanford, CA: Stanford University Press, 1992); Soraya De Chadarevian, *Designs for Life: Molecular Biology after World War II* (Cambridge: Cambridge University Press, 2002); Jon Agar, *Science in the Twentieth Century and Beyond* (Cambridge: Polity Press, 2013); David Edgerton, *Warfare State: Britain, 1920–1970* (Cambridge: Cambridge University Press, 2005). Internationalism in science has most often been through conferences, for instance, Elisabeth Crawford, Terry Shinn, and Sverker Sörlin (eds.), *Denationalizing Science: The Contexts of International Scientific Practice* (Dordrecht: Springer, 1993); Ronald E. Doel, Dieter Hoffmann, and Nikolai Krementsov, “National States and International Science: A Comparative History of International Science Congresses in Hitler’s Germany, Stalin’s Russia, and Cold War United States,” *Osiris* 20 (2005): 49–76; and the project led by Sven Widmalm, “The Scientific Conference: A Social, Cultural, and Political History” (2019–).

10. Malcolm Tight, *The Development of Higher Education in the United Kingdom since 1945* (Milton Keynes: Open University Press, 2009); A. H. Halsey, *The Decline of Donnish Dominion?* (Oxford: Clarendon, 1992), especially Ch. 6; Tamson Pietsch, *Empire of Scholars: Universities, Networks and the British Academic World, 1850–1939* (Manchester: Manchester University Press, 2013), Ch. 8.

11. Robert Maxwell, quoted in Joe Haines, *Maxwell* (London: McDonald & Co., 1988), p.139.

12. Noah Moxham and Aileen Fyfe, “The Royal Society and the Prehistory of Peer Review, 1665–1965,” *Historical Journal* 61 (2018): 863–89; Aileen Fyfe, “Editors, Referees and Committees: Distributing Editorial Work at the Royal Society Journals in the Late Nineteenth and Twentieth Centuries,” *Centaurus* 62 (2020): 125–40.

13. See, for instance, the geography of the Royal Society’s journal dissemination, Aileen Fyfe, “The Royal Society and the Non-Commercial Circulation of Knowledge,” in Martin Paul Eve and Jonathan Gray (eds.), *Reassembling Scholarly Communications: Histories, Infrastructures, and Global Politics of Open Access* (Cambridge, MA: MIT Press, 2020), pp.150–53. For the Royal Society’s engagement with British politics and with scientific diplomacy in the postwar period, see Jennifer Rose Goodare, “Representing Science in a Divided World: The Royal Society and Cold War Britain” (Ph.D., University of Manchester, 2013), esp. Ch. 2. See also Jeff Hughes, “Mugwumps? The Royal Society and the Governance of Post-War British Science,” in Don Leggett and Charlotte Sleigh (eds.), *Scientific Governance in Britain, 1914–79* (Manchester: Manchester University Press, 2016).

14. On Pergamon, see Haines, *Maxwell* (note 11), Ch. 5; Brian Cox, “The Pergamon Phenomenon 1951–1991: A Memoir of the Maxwell Years,” *Logos: Forum of the World Book Community* 9 (1998): 135–40; and Robert N. Miranda, “Robert Maxwell: Forty-Four Years as Publisher,” in Einar H. Fredriksson (ed.), *A Century of Science Publishing: A Collection of Essays* (Amsterdam: IOS Press, 2001). For postwar science publishing more generally, see Andrew Nash, Claire Squires, and Ian Willison (eds.), *The Cambridge History of the Book in Britain, Volume 7. The Twentieth Century and Beyond* (Cambridge: Cambridge University Press, 2019), Chs. 17–18; Einar H. Fredriksson, *A Century of Science Publishing: A Collection of Essays* (Amsterdam: IOS Press, 2001); H. Kay Jones, *Butterworths: History of a Publishing House* (London: Butterworth & Co., 1980), Ch. 8.

15. Derek Richter, “How Many More New Journals?” *Nature* 186 (1960): 18. This was a meeting of the Scientific Publications Council, sponsored by the Ciba Foundation.

16. “Code for the Publication of New Scientific Journals,” June 12, 1963, RS HF/160/2/7.

17. Dr. W. R. S. Garton (Imperial College, London), speaking at a meeting of the Scientific Publications Council, December 13, 1962, quoted in A. V. S. de Reuck, “Learned Societies as Publishers,” *Nature* 197 (1963): 426–7, 426.

18. John Coales (a Cambridge-based editor of a Pergamon journal), reporting the attitude of his colleagues in the 1950s, quoted in Haines, *Maxwell* (note 11), p.175. As Haines explains (pp.139–55), Maxwell was also widely disliked by British publishers due to his role in the liquidation of the wholesaling firm of Simpkin Marshall in 1955.

19. D. C. Martin, “The Royal Society’s Interest in Scientific Publications and the Dissemination of Information,” *Aslib Proceedings* 9 (1957): 127–41, 134–5, 140. On Martin, see Jeff Hughes, “Doing Diaries: David Martin, the Royal Society and Scientific London, 1947–1950,” *Notes and Records* 66 (2012): 273–94; Harrie Massey and Harold Thompson, “David Christie Martin, 1914-1976,” *Biographical Memoirs of Fellows of the Royal Society* 24 (1978): 390–407.

20. Frank Morley, *Self-Help for Learned Journals: Note Compiled for the Nuffield Foundation* (London: The Nuffield Foundation, 1963), p.10.

21. The results of the National Science Foundation’s survey of journal editors were reported in Robert Tumbleson and Helen L. Brownson, “Survey of Operations and Finances of Scientific Journals,” *Science* 119 (1954): 357–9, quote at 357. On page-charges in the United States, see Tom Scheiding, “Paying for Knowledge One Page at a Time: The Author Fee in Physics in Twentieth-Century America,” *Historical Studies in the Natural Sciences* 39 (2009): 219–47, and Marianne Noel, “Building the Economic Value of a Journal in Chemistry. The Case of the Journal of the American Chemical Society (1879–2010),” *Revue française des sciences de l’information et de la communication* (2017). https://doi.org/10.4000/rfsic.3281

22. On the Nuffield Foundation, see Ronald W. Clark, *A Biography of the Nuffield Foundation* (London: Longman, 1972). The main output of the project was Frank Morley’s *Self-Help* (note 20). The committee minutes survive in the Nuffield Foundation archive. Related material survives in the archives of the various learned societies involved, including the Royal Society, Royal Society of Chemistry, Royal Geographical Society, and Royal Anthropological Institute.

23. Morley, *Self-Help* (note 20), p.9.

24. Aileen Fyfe, “Journals, Learned Societies and Money: Philosophical Transactions, Ca. 1750–1900,” *Notes and Records of the Royal Society* 69 (2015): 277–99; Fyfe, “The Non-Commercial Circulation of Knowledge” (note 13).

25. Rayleigh to the Treasury (note 1). See also Fyfe, “Journals, Learned Societies and Money” (note 24).

26. The Royal Society lobbied the government: “The Publication of Results of Scientific Research in the United Kingdom” (1947?), p.5, uncatalogued in RS OM3. On the growth of the Royal Society’s political engagement in the mid-twentieth century, see Peter Collins, *The Royal Society and the Promotion of Science since 1960* (Cambridge: Cambridge University Press, 2015).

27. Morley, *Self-Help* (note 20), p.9.

28. On the difficulties facing UK university presses, see David McKitterick, *A History of Cambridge University Press: Volume 3, New Worlds for Learning, 1873–1972* (Cambridge; New York: Cambridge University Press, 2004) and Wm Roger Louis, *The History of Oxford University Press, Volume III: 1896 to 1970* (Oxford; New York: Oxford University Press, 2013). For Cambridge University Press’s (CUP) renegotiation with the Royal Society, see letter from R. J. L. Kingsford (of CUP) to RS, November 26, 1953, in RS OM/61(53).

29. Tumbleson and Brownson, “Operations and Finances” (note 21), 357.

30. Minutes of the Learned Journals Scientific Advisory Committee (henceforth LJ-SAC), April 15, 1955, Nuffield Foundation archives (henceforth, Nuffield) L.J.1. Also, Clark, *The Nuffield Foundation* (note 22), pp.135–7.

31. Clark, *The Nuffield Foundation* (note 22), Chs. 1–2.

32. Collins, *The Royal Society since 1960* (note 26), p.236.

33. On the conference, see Brian Vickery, “The Royal Society Scientific Information Conference of 1948,” *Journal of Documentation* 54 (1998): 281–3; and Hughes, “Mugwumps” (note 13).

34. Production costs were reported in the annual accounts and published in the volumes of the *Year-Book of the Royal Society*. Number of published pages, supplied by Royal Society publishing team.

35. “Consideration of Estimated Deficit on RS Publications Account,” November 4, 1953, uncatalogued memo in RS OM3.

36. R. J. L. Kingsford (of CUP) to RS, November 26, 1953, in RS OM/61(53). On CUP, see McKitterick, *Cambridge University Press 3* (note 28), Ch. 17.

37. Minutes of officers meeting, November 30, 1953, RS OM/62(53).

38. Sales income from publishing was reported in the annual accounts and published in the volumes of the *Year-Book of the Royal Society*.

39. Martin, “Royal Society’s Scientific Publications” (note 19), 135.

40. D. C. Martin (for the Royal Society) to the Chemical Society, January 15, 1957, in Minutes of Council, January 17, 1957, archive of the Chemical Society (now part of the Royal Society of Chemistry) C.P./4(57), p.5.

41. Martin, “Royal Society’s Scientific Publications” (note 19), 136.

42. LJ-SAC, April 15, 1955, Nuffield L.J.1. A further £20,000 was allocated to the equivalent advisory committee in partnership with the British Academy.

43. Ibid.

44. For the new Royal Society attitude to the grant process, see Ad-hoc Committee on the Economics of Learned Journals, November 20, 1957, RS CMB/148b.

45. Unconfirmed notes of meeting on December 8, 1953, quoted in “Notes on an Informal Meeting to Discuss Publication and Sales Promotion of Scientific Journals,” held at the Royal Society, July 19, 1956; copy from the archives of the Royal Anthropological Institute (hereafter RAI), 40/2/13.

46. Clark, *The Nuffield Foundation* (note 22), p.136.

47. Lusty Report, quoted in Morley, *Self-Help* (note 20), p.10 (“no special interest”); LJ-SAC, April 15, 1955, Nuffield L.J.1.

48. The Chemical Society was already discussing the need to “improve the financial position” of its journal; see Minutes of Council, January 17, 1957, Chemical Society archive, C.P./4(57), p.5. Later that year, the Chemical Society contributed to a committee set up by the Royal Society to investigate problems with chemistry journals; see Minutes of Council, August 9, 1957, Chemical Society archive C.P./41(57), p.46, and papers of the “Problems of Publication in Chemistry” Committee, RS CMB/93. For the wider context, see David Hardy Whiffen and Donald H. Hey, *The Royal Society of Chemistry: The First 150 Years* (London: The Royal Society of Chemistry, 1991).

49. His report does not appear to survive in the Nuffield or Royal Society archives, but it was discussed by committees in both organizations and was later quoted in Morley, *Self-Help* (note 20), passim.

50. Morley, *Self-Help* (note 20), p.10. This was not strictly true, because some of the larger societies – including the Royal Society, Institute of Physics, and Chemical Society – did have paid editorial staff.

51. Ibid., p.11. On the “Experimental Pro-Printing Plant” that Martin established at the Royal Society, see Memorandum (undated, perhaps December), 1951, in RS OM/66(51).

52. LJ-SAC, March 16, 1956, Nuffield L.J.2. The Association of Learned and Professional Society Publishers would not be formed until 1972, and the Society Publishers’ Coalition in 2019.

53. LJ-SAC, February 24, 1959, Nuffield L.J.13.

54. LJ-SAC, March 16, 1956, Nuffield L.J.2.

55. Charles Chadwyck-Healey, *Publishing for Libraries: At the Dawn of the Digital Age* (London: Bloomsbury, 2020), p.15.

56. Jones, *Butterworths* (note 14), pp.130–39. Maxwell created Pergamon from the remains of the Butterworth-Springer collaboration.

57. LJ-SAC, March 25, 1957, Nuffield L.J.3.

58. Ibid., and LJ-SAC, May 29, 1957, Nuffield L.J.4.

59. “Grants for Learned Journals: Dr F.V. Morley,” *Nature* 180 (1957): 465.

60. LJ-SAC, March 25, 1957, Nuffield L.J.3.

61. He was initially appointed for three years at £1,500 pa, LJ-SAC, March 25, 1957, Nuffield L.J.3. His contract was extended for at least a further year in 1960, but the archival record ends at this point: LJ-SAC, October 5, 1960, Nuffield L.J.18. Morley delivered an internal “final” report in 1960, but this does not appear to survive in the Nuffield archive. A 1963 draft of *Self-Help* survives in the Royal Society archive (RS HF/1/17/3/2) but is largely the same as the published version.

62. “Grants for Learned Journals” (note 59).

63. I have found only seven examples where financial assistance was requested and denied without advice being explicitly offered instead (or in the interim); in all but one case, the reason was that the specific project for which funding was requested was deemed to be outside the committee’s remit, not because the applicant was ineligible. The Queckett Microscopical Club is the only organization that appears to have been deemed ineligible.

64. “Grants for Learned Journals” (note 59).

65. LJ-SAC, May 29, 1957, Nuffield L.J.4.

66. Edward Salisbury, in Morley, *Self-Help* (note 20), p.[5].

67. LJ-SAC, December 4, 1957, Nuffield L.J.8.

68. LJ-SAC, October 9, 1959, Nuffield L.J.15.

69. LJ-SAC, October 28, 1957, Nuffield L.J.7.

70. The arrangements were found to be “reasonable,” LJ-SAC, February 24, 1959, and June 23, 1959, Nuffield L.J.13 and L.J.14.

71. Morley, *Self-Help* (note 20), pp.42–4.

72. For instance, Institute of Navigation / John Murray, LJ-SAC, September 8, 1958, Nuffield L.J.12; and British Bryological Society / Cambridge University Press, LJ-SAC, February 24, 1959, Nuffield L.J.13.

73. LJ-SAC, June 23, 1958, Nuffield L.J.11.

74. LJ-SAC, February 24, 1959, Nuffield L.J.13. A request to support the indexing of *The Observatory* met a similar response, LJ-SAC, June 23, 1959, Nuffield L.J.14.

75. Morley, *Self-Help* (note 20), p.18. The public account of the anonymous society fits with Morley’s report of changes at the British Glaciological Society, LJ-SAC, February 17, 1958, Nuffield L.J.9. The South London Natural History Society was given the same advice later that year, LJ-SAC, June 23, 1958, Nuffield L.J.11.

76. Morley, *Self-Help* (note 20), pp.14–15.

77. Ibid., p.25 (quoting Lusty) and pp.37–8.

78. Lusty report, discussed at LJ-SAC, March 16, 1956, L.J.2.

79. Haines, *Maxwell* (note 11), p.175.

80. I have identified “Society A” as the Chemical Society on the grounds that the rises in subscription prices for 1957/8 match those the society made that year: see Minutes of Council, March 14, 1957, Chemical Society archive C.P./18(57), p.17.

81. Morley, *Self-Help* (note 20), pp.24–6.

82. Ibid., p.25.

83. LJ-SAC, October 28, 1957, Nuffield L.J.7 (“more effective” and “spending a little”); March 2, 1960, Nuffield, L.J.16 (“more elaborate”).

84. Morley, *Self-Help* (note 20), p.22.

85. Ibid., p.26.

86. LJ-SAC, October 28, 1957, Nuffield L.J.7.

87. LJ-SAC, September 23, 1957, Nuffield L.J.6.

88. Morley, *Self-Help* (note 20), pp.26–7.

89. In contrast, Maxwell developed links with the Soviet and Chinese authorities, aiming to make Pergamon a conduit for scientific communication (in both directions) across the Iron Curtain. See Haines, *Maxwell* (note 11), pp.159–62.

90. According to the Institute for Navigation, which also believed that the British market had reached “saturation point,” LJ-SAC, June 23, 1958, Nuffield, L.J.11.

91. On the growing links between academia in the United States and the British world, see Pietsch, *Empire of Scholars* (note 10), Ch. 8, especially pp.177–8.

92. LJ-SAC: October 28, 1957, Nuffield L.J.7; April 14, 1958, Nuffield L.J.10; and May 18, 1960, Nuffield L.J.17.

93. LJ-SAC, September 8, 1958, Nuffield L.J.12.

94. LJ-SAC, March 2, 1960, Nuffield L.J.16.

95. LJ-SAC, September 23, 1957, Nuffield L.J.6.

96. The geology list was in proof by fall 1959, and there were plans for lists for mathematics, physiology, botany, and zoology, see LJ-SAC, October 9, 1959, Nuffield L.J.15. By 1963, three lists (geology, mathematics, zoology); appear to have been issued; see Morley, *Self-Help* (note 20), p.30.

97. LJ-SAC, December 4, 1957, Nuffield L.J.8.

98. Morley, *Self-Help* (note 20), p.35.

99. LJ-SAC, February 17, 1958, Nuffield L.J.9.

100. Morley, *Self-Help* (note 20), pp.35–6. Shell-Mex was only considering spending £1,200 and £2,400 a year on advertising; LJ-SAC, February 24, 1959, Nuffield L.J.13. On lack of interest, see LJ-SAC, October 9, 1959, Nuffield L.J.15.

101. Morley, *Self-Help* (note 20), p.36. Having failed to get the geological societies to cooperate, Morley instead tried to secure Shell-Mex advertising for the select list of geological periodicals, LJ-SAC, May 18, 1960, Nuffield L.J.17.

102. Morley, *Self-Help* (note 20), pp.33–4.

103. LJ-SAC, September 23, 1957, Nuffield L.J.6.

104. *Year-Book of the Royal Society*: 1959, p.208; and 1960, p.219.

105. LJ-SAC, February 24, 1959, Nuffield L.J.13. For the repayment, see LJ-SAC, October 9, 1959, Nuffield L.J.15 and March 2, 1960, Nuffield L.J.16.

106. LJ-SAC, May 18, 1960, Nuffield L.J.17.

107. With the Linnean microfiches, the Nuffield committee made it explicit that any profits must be applied to the journal; see LJ-SAC, February 24, 1959, Nuffield L.J.13.

108. On the Institute of Physics, see Tom Scheiding, “Paying for Knowledge” (note 21); Stephen R. Geiger, “Income from Authors,” *IEEE Transactions on Professional Communication* PC-10 (1975): 97–102; and Tumbleson and Brownson, “Operations and Finances” (note 21), 359.

109. The American Chemical Society adopted page charges in 1963; see Noel, “Building the Economic Value of a Journal in Chemistry” (note 21).

110. Except for being very briefly floated at the Chemical Society in 1957, before being dismissed as being potentially “contrary to the Society’s Charter,” see “Journal Finances,” p.3, in Minutes of Council of the Chemical Society, Appendix B to C.P./15/(57).

111. LJ-SAC, October 5, 1960, Nuffield L.J.18.

112. The president’s copy of the paperwork for that June 12, 1963, meeting is in the Howard Florey papers, RS HF/1/17/3/2/-. See also papers of the Conference of Editors, 1964–8, RS CMB/150.

113. Edward Salisbury, in Morley, *Self-Help* (note 20), p.[5].

114. Morley, *Self-Help* (note 20), p.12.

115. Marian Smith to Frank Morley, January 23, 1958, RAI 150/6/29.

116. LJ-SAC, June 23, 1959, Nuffield L.J.14, quoting Marian Smith to Frank Morley, June 11, 1959, RAI 150/6/42. By the early 1960s the institute’s two journals were both generating a surplus, though its overall publishing activities continued to run at a deficit; see *Annual Report and Accounts of the Royal Anthropological Institute of Great Britain and Ireland for the Year Ending 31 December 1963*, schedule III.

117. Morley, *Self-Help* (note 20), p.9.

118. See Larivière, Haustein, and Mongeon, “The Oligopoly of Academic Publishers” (note 3).

119. See, for instance, Christine Baldwin, “What Do Societies Do with Their Publishing Surpluses? ALPSP and Blackwell Survey 2004,” (Worthing, UK: ALPSP, 2004), and the wide variety of activities reported in Collins; *The Royal Society since 1960* (note 26).

